# Components Influencing Nurses’ Employability in Organisational Transitions: A Mixed‐Methods Study

**DOI:** 10.1155/jonm/4336429

**Published:** 2026-04-17

**Authors:** Tamaki Isobe, Yukie Takemura, Keiko Kunie

**Affiliations:** ^1^ Department of Nursing Administration, Division of Health Sciences and Nursing, Graduate School of Medicine, The University of Tokyo, Tokyo, Japan, u-tokyo.ac.jp; ^2^ Nursing Department, The University of Tokyo Hospital, Tokyo, Japan, u-tokyo.ac.jp; ^3^ Department of Nursing Sciences, Graduate School of Human Health Sciences, Tokyo Metropolitan University, Tokyo, Japan, tmu.ac.jp

**Keywords:** employability, interorganisational mobility, mixed-methods, nursing workforce, recruitment, sustainable careers

## Abstract

**Background:**

Sustainable careers are becoming increasingly important in nursing, as workforce shortages and high turnover rates challenge global healthcare systems. Employability, which refers to the skills that enable individuals to obtain and maintain employment, is essential for nurses undergoing interorganisational transitions.

**Aim:**

This study examined the components of nurses’ employability and evaluated their relative importance in recruitment decisions across various healthcare settings in Japan.

**Methods:**

The study employed a mixed‐methods approach. It comprised a qualitative phase, involving semistructured interviews with nurse recruiters to identify key employability components, followed by a quantitative phase that employed conjoint analysis to evaluate the relative significance of these components. The participants included recruiters from hospitals, long‐term care facilities and homecare nursing agencies.

**Results:**

Seven key employability components were identified: long‐term retention potential, practical nursing skills, an optimistic approach to challenges, willingness to learn about nursing as a profession and ideology, relationship‐building abilities, commitment to organisational improvement and intrinsic motivation for nursing. The conjoint analysis revealed both common and setting‐specific priorities. Hospitals valued “Practical skills”, “Establishing relationships” and “Motivation for nursing”, irrespective of bed size. Homecare nursing agencies emphasised practical skills, relationship building and nurses’ motivation. In contrast, long‐term care facilities prioritised interpersonal relationships with others. Across all the settings, clarity regarding the reasons for and objectives of previous job changes was critical for recruitment decisions.

**Conclusion:**

This study enhances our understanding of how employability is conceptualised and evaluated in nursing recruitment. Employability is not limited to individual competencies but reflects the alignment between nurses’ professional orientation and organisational values, supporting sustainable nursing careers.

**Implications for Nursing and/or Health Policy:**

This study underscores the importance of tailoring employability to meet the unique needs of healthcare facilities. By enhancing employability, nurses can achieve their desired career goals and adapt to evolving organisational needs, thereby benefiting both individuals and healthcare systems.

## 1. Introduction

Recognised globally as a critical health emergency, nursing shortages require immediate attention. With the recent ageing of populations worldwide, the demand for nursing care is expected to increase across various healthcare settings, from acute care to recovery and long‐term care [1, 2]. Addressing this demand requires ensuring a sustainable nursing workforce in which licensed nurses can adapt flexibly to their work settings and continue to work in the long term, in response to life events and career goals.

For nurses, sustainable careers involve maintaining meaningful employment, whether by staying in their current roles or transitioning to new ones that better align with their personal aspirations. Importantly, the concept of a sustainable career is intended to address the concerns of both employees and employers jointly. De Vos and Van der Heijden [3] characterised individual sustainable careers based on four attributes: (1) alignment of work with the individual’s strengths, interests and values; (2) ongoing learning and renewal; (3) security through employability and (4) work‐life fit over the life course. Crucially, these attributes directly benefit organisations, as they correspond closely to the core objectives of talent management: (1) maximising returns on human capital, (2) maintaining and updating organisational competencies, (3) achieving stability through adaptability and (4) fostering commitment and retention. However, successful long‐term alignment depends on the fit between nurses’ skills and competencies and the requirements of healthcare organisations. Organisations must recruit nurses with the appropriate skill sets and retain them for operational stability [4]. For nurses, understanding organisational expectations and enhancing employability are essential for career continuity and long‐term satisfaction.

Employability, defined as the continuous ability to secure, maintain and create work through the optimal use of competencies, is a core element of a sustainable career [5]. It encompasses internal factors, such as skills, motivation and adaptability, and external factors, such as organisational support and workplace conditions [6, 7]. By cultivating employability, nurses can better align with organisational needs, contributing to their own career satisfaction, as well as improving retention rates and the overall effectiveness of the healthcare workforce.

Despite its importance, employability in nursing remains underexplored, especially concerning interorganisational mobility. Previous studies have predominantly focused on specific populations, such as newly graduated nurses [8, 9] or older nurses facing job insecurity [10, 11]. However, the factors influencing employability among non‐new graduate nurses undergoing interorganisational transitions, particularly across diverse healthcare settings, have received limited attention.

This research gap is critical, given the existing mismatches between the competencies sought by healthcare organisations and the abilities of available nurses, despite the overall nursing shortage. In Japan, nurses often transition to smaller hospitals, long‐term care facilities and homecare services when changing jobs, as many begin their careers in teaching or acute care hospitals, where education and training systems are well established, and later move to more specialised or smaller facilities to pursue their professional interests or achieve a better work‐life balance [12].

However, the specific qualities and characteristics of nurses sought by different types of healthcare facilities remain unclear. Additionally, the rapidly ageing and declining working‐age population have exacerbated nursing shortages. Despite numerous job openings and seekers, mismatches between positions and nurses’ qualifications persist [13]. Addressing these challenges requires a deeper understanding of the factors influencing employability during workplace transitions, particularly how they vary across healthcare settings.

Therefore, this study aims to explore the key components of employability and evaluate their relative importance in recruitment decisions in various healthcare settings. Specifically, the objectives were twofold: (1) to identify the components that constitute nurses’ employability and (2) to assess the relative importance of these components in the recruitment process. By identifying the factors that enhance employability, this study seeks to contribute to strategies that support both individual career sustainability and the broader stability of the healthcare workforce.

## 2. Materials and Methods

### 2.1. Design

This study employed a mixed‐methods approach, using a partially mixed sequential dominant‐status design [14]. A qualitative descriptive methodology was used in the initial phase, followed by quantitative methods and conjoint analyses in the second phase.

The initial phase aimed to elucidate the components of nurses’ employability when transitioning between workplaces, which suggest that healthcare facilities are deemed essential for recruiting nurses. The second phase aimed to identify the differences in the weighting of employability constructs by facility type. This will provide clues as to which competencies nurses should supplement, depending on the type of facility in which they wish to work.

“Nurse’s employability” was defined as “the abilities, conditions and factors that contribute to employment decisions made by recruiters when considering whether to hire a nurse moving from one healthcare facility to another”.

### 2.2. Qualitative Descriptive Study: Data

We conducted a qualitative inductive study using qualitative content analysis during the initial phase [15]. Data were collected through semistructured interviews with nurse recruiters in Japan working in healthcare facilities and job placement agencies in Japan. Most nurses secure jobs by directly applying to healthcare facilities or job agencies, making the participants well‐versed in the recruitment process. The selection criteria for participants included having at least 2 years of experience in conducting interviews or 5 years in the nursing recruitment field. For job placement agencies, a minimum of 1 year of experience in placing with facilities was required. The study included both licensed and unlicensed individuals who met the inclusion criteria.

We first employed the opportunity method to recruit participants by presenting the study to facility heads who subsequently recommended individuals who met the criteria. The researchers contacted these candidates via email to discuss the research and its ethical considerations, and all eight agreed to participate.

Each participant completed a single interview. Recruiters from healthcare facilities were asked about the factors that encouraged them to hire and those that made them hesitant to hire particular candidates. For job placement agencies, the questions focused on the characteristics of the applicants who were more or less likely to be hired by healthcare facilities. The research team conducted interviews, both in person and online, and audio‐recorded them.

The interviews averaged 69.5 min in length, with durations ranging from 54 to 88 min. Interviews were conducted between March and August 2020 by the first author, who was previously unfamiliar with the participants. The interviewer, a registered nurse with a master’s degree in nursing, conducted all the interviews.

### 2.3. Qualitative Descriptive Study: Data Analysis

Data were analysed inductively by applying the qualitative content analysis described by Graneheim and Lundman [15]. Verbatim transcripts were created from the interview data. Then, through repeated reading of the transcript, the text was divided into meaning units which described nurses’ competencies, hiring conditions and related factors, which were then labelled with codes. Then, by analysing the condensed meaning of the context, codes representing similar characteristics were grouped into subcategories and categories that described the components of nurses’ employability. We confirmed that adding data from another participant did not generate new subcategories. Consequently, themes that encompassed all categories were identified. The research software MAXQDA 2020 was used to manage and analyse the data.

### 2.4. Qualitative Descriptive Study: Rigour and Trustworthiness

We adopted a rigorous study design, analysis and writing methods aligned with the standards and framework guidelines of previous qualitative research [15–18]. To enhance credibility, participants with diverse demographics and experiences were included. Several researchers specialising in nursing management repeatedly discussed and revised the appropriateness of measurement units, codes and categories [15]. To ensure confirmability, the research team maintained an audit trail and engaged in reflexive discussions to minimise individual bias. To enhance transferability, detailed descriptions of the research context, participant characteristics and data collection procedures were provided, allowing readers to assess the applicability of the findings in other settings. This study was conducted in accordance with the Consolidated Criteria for Reporting Qualitative Studies (COREQ) checklist for qualitative research [19].

### 2.5. Conjoint Analysis: Procedure

A conjoint analysis was applied in the second phase to evaluate the importance of various factors affecting nurses’ employability. In this method, the elements influencing a product’s value are called “attributes”, and their specific variations are termed “levels”. We estimated the utility score for each factor by selecting one level from each attribute and presenting the combined profiles to the respondents. This score indicates significance in decision‐making and allows for potential trade‐offs [20].

Healthcare facilities typically consider the multiple aspects of each applicant during the hiring process. Conjoint analysis helps to clarify the relative importance of these factors, reflecting the practical hiring decision‐making process.

Based on the first phase, we developed virtual profiles for job‐seeking nurses by identifying nine key factors: three at three levels and seven at two levels (Table 1). We also employed an experimental design methodology to facilitate profile creation using an orthogonal array table from the IBM SPSS Conjoint ORTHOPLAN. This approach ensures a systematic and structured assessment of the attributes involved in profile construction. To ensure internal validity, we incorporated two holdout cases, following Rao’s recommendation [20], resulting in 18 virtual profiles. These profiles provide a structured framework for analysing nurse recruiters’ preferences and decision‐making processes.

**TABLE 1:** Example of virtual profiles.

Attributes (number of levels)	Profile 1	Profile 2	…	Profile 18
Reasonability for changing facility (3)	Unknown	With	…	Without
Experience of sustained employment in one organisation (2)	Without	With	…	With
Readiness to work (2)	With	With	…	With
Practical nursing skill (3)	Poor	Excellent	…	Moderate
Problem‐solving orientation (2)	Negatively	Positively	…	Positively
Alignment towards nursing (2)	With	With	…	With
Establishing relationships with others (2)	With	With	…	Without
Commitment to organisation (2)	With	With	…	Without
Motivation for nursing (2)	With	With	…	Without

*Note:* Levels of attributes in each profile were combined according to an orthogonal array table. The ellipsis (“…”) indicates intermediate profiles omitted for brevity.

Participants first categorised candidate profiles into three groups: “Preferred for hire”, “Acceptable for hire” and “Not preferred for hire”. After this initial grouping, they ranked the candidates within each group based on their hiring preferences. Finally, each respondent provided a complete ranking of all 18 candidate profiles, from 1 (*most preferred*) to 18 (*least preferred*), based on their overall hiring preferences. We collected data on respondents’ age, sex, job title, position, tenure, qualifications and the type and functionality of the facility they worked in.

A pilot test was conducted by a researcher specialising in nursing administration and with experience in nurse recruitment. This study aimed to confirm the clarity and answerability of the questions. Minor wording revisions were made to the instructions based on participants’ feedback.

### 2.6. Conjoint Analysis: Data

Self‐administered questionnaires were used for data collection. The survey targeted nurse recruiters working at healthcare facilities throughout Japan. They were stratified and randomly selected according to the facility type: hospitals (with more than 200 beds), hospitals (with less than 200 beds), clinics, homecare nursing agencies and long‐term care facilities. Random sampling was conducted using several publicly available online databases that were considered to provide broad coverage for each type of healthcare facility [21–23]. Participants were selected based on their employment in the target facilities and their involvement in nurse recruitment decisions.

We sent the survey form requests, URLs and QR codes to facility managers and asked them to invite up to two individuals who met the inclusion criteria to participate in the survey. The study was conducted over a period of 2 weeks, from September to October 2020.

This study followed the Good Research Practices for Conjoint Analysis Task Force guidelines set by the International Society for Pharmacoeconomics and Outcomes Research (ISPOR) [24]. The study was designed and reported in accordance with the “Strengthening the Reporting of Observational Studies in Epidemiology (STROBE)” statement [25].

### 2.7. Conjoint Analysis: Data Analysis

Descriptive statistics were calculated for participants’ individual and facility attributes. The respondents were then stratified into five categories based on the type of facility to which they were affiliated: hospitals (divided into those with more than 400 beds, 200–399 beds and less than 200 beds), clinics, homecare nursing agencies and long‐term care facilities. We used the ranked data obtained from respondents to estimate the average relative importance and partial utility of each attribute for each facility type. The attribute levels were treated as nominal scales, leading to the application of DISCRETE or DISCRETE‐LESS models in the estimation process. All statistical analyses were performed using IBM SPSS Statistics Ver. 27 and IBM SPSS Conjoint, with a significance level of 5%.

### 2.8. Ethical Considerations

The Research Ethics Committee of the Faculty of Medicine and The University of Tokyo approved both studies (approval numbers: 2019273NI and 2020165NI). We ensured participant confidentiality, voluntary participation and absence of coercion. Before the survey commenced, participants were provided participants with a written explanation of the study, and their informed consent was obtained.

## 3. Results

### 3.1. Qualitative Descriptive Study: Theme and Components of Nurses’ Employability

Eight people participated in the interviews: five nurse recruiters from healthcare facilities and three from job placement agencies (Table 2).

**TABLE 2:** Characteristics of qualitative interview participants (*n* = 8).

		*n* or mean
Medical care facility recruiters		5
Facility type	Hospital (over 400 beds)	1
	Hospital (200–399 beds)	2
	Homecare nursing agency	1
	Long‐term care facility	1

Position	Facility director equivalent	1
	Nursing director equivalent	2
	Deputy director equivalent	1
	Nurse manager equivalent	1

Age range	50–59	3
	40–49	1
	30–39	1

Years in the current facility	12.7 (range: 2.4–23)	

Years in nurse recruitment duties	7.7 (range: 1.6–18)	

Recruiting agency		3
Age range	60–69	2
	40–49	1

Years in current job	8.7 (range: 5–15)	

We extracted seven categories and 19 subcategories to represent the components of nurses’ employability (Table 3). The theme encompassing the seven categories was *the ability to work together on the nursing care that the organisation is trying to provide*. This theme suggests that recruiters look for applicants who can seamlessly integrate into the organisation and effectively fulfil the nursing role to contribute positively to their employment. They expect the applicants to fit in with the organisation and take on the required nursing role to contribute to employment. These seven categories are described in the following order: The category names are indicated by [ ], the subcategory name by “ ” and the code name by ‘ ’ in the text and tables.

**TABLE 3:** Components of nurse’s employability.

Category	Subcategory	Code examples
1. Expected retention in the organisation	1.1. Previous job changes have been for a clear purpose or tenable reasons	If they “Have a clear vision of what they want to do at the facility” and “Can properly explain the reasons for changing jobs to the understanding of the recruiter” they are considered ideal candidates. Conversely, it gives a negative impression if they have “Repeatedly changed jobs because of short‐sightedness”.
	1.2. Experience of sustained employment in one organisation	If they “Have worked at one facility for a long time”, “Number of facilities experienced” and “Left after less than a year, irrespective of the reason”.
	1.3. Prepared and ready to work for the long term	If they “Have a desire to do well in the new job” and “Understand the changes to their lives and the impact on their families by returning to work”.

2. Expected practical skills	2.1. Ability to plan and practice nursing care autonomously	If they “Have the skills to conduct basic day‐to‐day tasks”, “Have experienced caring for in‐ward patients throughout the year” and “Can plan and prioritize daily plans for the day on their own in practice”.
	2.2. Ability to make clinical judgements	If they “Can observe the holistic view of clients” and “Assess and judge the patient’s condition”.
	2.3. Willingness to learn and improve practical skills	If they can “Learn new things honestly in a new place”, “Use their thinking skills”, and “If they are taught from the basics in the department, they will not be able to keep up”.
	2.4. Ability to undertake a leadership role	If they “Can take on roles as required by years of experience”, “Have a full range of experience in leadership and student mentoring” and “Can organise team opinions in committee activities”.

3. Positivity when faced with challenges	3.1. Taking a proactive perspective on challenges	If they “Blame the organization for the reason they want a job change” and “Tried resolving dissatisfaction by changing jobs” are considered as negative aspects.
	3.2. Drawing lessons from any experience	If they ‘Can talk about what they have learned from their previous workplaces’ and ‘Reflect on past experiences and learn from them’.

4. Alignment with the organisation’s nursing philosophy	4.1. Attitude to understand the nursing required by the organisation	If they “Can switch from a medical‐centered perspective to a life‐centered perspective” and “Understand the different functions and roles of nurses”.
	4.2. Match between the nursing they want to provide and the organisation’s nursing	If they “Are willing to improve the nursing care they provide” and “Can narrate patient‐ and family‐centered episodes”.

5. Establishing a good relationship with the people involved	5.1. Respect and communicate with patients/clients and their families	If they “Can communicate and take the time to engage with patients/users” and “Can sincerely dedicate themselves to patients, conveyed through the episodes”.
	5.2. Work while communicating with others	If they “Can consult the appropriate person, such as a senior colleague or supervisor, when they need help” and “Can communicate with people across occupational boundaries”.
	5.3. Fit in as a member of the organization	If they “Accept different ways of conducting their duties and propose better modifications or improvements”.

6. Commitment to the organisation	6.1. Awareness of contribution to the organisation	If they “Can establish a give‐and‐take relationship with the organisation”, “Can talk about how one’s experience can be applied in the organisation” and the participants know “It is a major challenge to get employees who contribute to the hospital”.
	6.2. Attitude toward participating in the organisation and sharing its direction	If they “Can share the organization’s vision” and “Can participate honestly in the education system, understanding that they are part of the organization”.

7. Motivation for nursing	7.1. Has a passion for nursing	If they “Have a clear idea of the nursing they want to provide” and “Can write about their thoughts on nursing”.
	7.2. Can work sincerely in nursing	If they “Can proactively and independently provide the best possible care” and “Can state what care they want to provide their patients and their skill development steps with proofs”.
	7.3. Has a desire to improve as a nurse	If they “Are highly motivated to learn” and “Are willing to develop their nursing career”.

Category 1 (Expected retention in the organisation) indicates that the applicant would continue working at the facility. It comprises three subcategories. The first was “Previous job changes have been for a clear purpose or tenable reasons”. The decision on nurse hiring was affected by whether the applicant’s reasons for changing or leaving jobs in the past were unavoidable or acceptable to the recruiter, or if the applicant had changed jobs with a clear purpose. The second subcategory, “Experience of sustained employment in one organisation”, indicates that working for a particular organisation for a consistent period leads to employment. The third subcategory, “Prepared and ready to work for the long term”, indicates that applicants were expected to be psychologically and environmentally ready for work.

Category 2 (Expected practical skills) indicates that the applicant met the practical nursing skills level required by the organisation. If an applicant does not yet meet the required level at the time of employment, there is an expectation that they will develop the necessary skills after employment. The first subcategory was “Ability to plan and practice nursing care autonomously”, indicating that the applicant could provide day‐to‐day nursing care independently. Next was the “Ability to make clinical judgements”, suggesting that the applicant is required to observe and clinically judge patients/clients at the facility level. Then, the “Willingness to learn and improve practical skills” indicates the applicants’ willingness to make proactive efforts to upgrade their practical skills required by the organisation. Lastly, the category also includes “Ability to undertake a leadership role”.

Category 3 (Positivity when faced with challenges) indicates that when faced with challenges, even those partly stemming from organisational factors, the applicant recognises their own role in addressing them and demonstrates a proactive attitude towards problem‐solving. The subcategory “Taking a proactive perspective on challenges” suggests that the applicant recognises their own role in addressing issues rather than attributing causes or dissatisfaction to others. The “Drawing lessons from experience” subcategory signifies that the applicant learns from both positive and negative experiences.

Category 4 (Alignment with the organisation’s nursing philosophy) indicates the applicant’s attitude towards understanding what nursing care the facility provides. The subcategory “Attitude to understand the nursing required by the organisation” indicates that the applicant is willing to understand the differences in perspectives and roles between their nursing experiences and the nursing required by the facility. Particularly in homecare nursing agencies and long‐term care facilities, “Can switch from a medical‐centred perspective to a life‐centred perspective” and “Understand the different functions and roles of nurses” are emphasised. The subcategory “Match between the nursing they want to provide and the organisation’s nursing” also highlights the alignment of the applicant’s perspective on nursing with the organisation’s values.

Category 5 (Establishing a good relationship with the people involved) indicates that the applicant should be able to integrate into the organisation after employment and build good relationships while communicating with patients/users, their families, nursing staff and other colleagues. The subcategories of “Respect and communicate with patients/clients and their families”, “Work while communicating with others” and “Fit in as a member of the organisation” are included.

Category 6 (Commitment to the organisation) indicates that the applicant should understand the organisation’s vision and future directions and be willing to contribute proactively to the organisation. The subcategory “Awareness of contribution to the organisation” included codes, such as “Can talk about how one’s experience can be applied in the organisation”. Additionally, the challenges in identifying such nurses were noted. The subcategory “Attitude towards participating in the organisation and sharing its direction” represented that the applicant demonstrated proactive involvement and shared its direction.

Category 7 (Motivation for nursing) indicates that the applicant should possess passion, commitment and a desire to continually improve as a nurse. The subcategories “Has a passion for nursing”, “Can work sincerely in nursing” and “Has a desire to improve as a nurse” are included in this category.

### 3.2. Conjoint Analysis: Participant Characteristics

Researchers distributed seven thousand respondent IDs to 3500 target facilities, and 222 respondents answered all the questions. A total, 220 respondents were included in the analysis, representing a valid response rate of 3.1%. Two types of responses were excluded: those showing an obviously untruthful response pattern (e.g., selecting the same option for all items) and duplicate responses submitted under the same respondent ID. The valid response rates by facility type were 14.6% for hospitals (≥ 200 beds), 6.6% for hospitals (< 200 beds), 1.4% for clinics, 3.9% for homecare nursing agencies and 5.0% for long‐term care facilities.

Table 4 presents the respondents’ attributes and institutional characteristics. Most of the respondents were nurses (76.4%), followed by administrative staff (12.7%), others (7.7%) and doctors (2.3%). The most common job title was “Director of Nursing and Human Resources” (44.1%), followed by “Deputy Director” (17.3%). Respondents were primarily from hospitals with 200–399 beds (25.9%), followed by those with fewer than 200 beds (20.9%) and hospitals with more than 400 beds (20.5%). The job types of the respondents varied according to the type of facility. In clinics, 50% of the respondents were doctors, whereas in homecare agencies, 85.7% were nurses. In long‐term care facilities, 42.9% were care staff, 37.0% were administrative staff and 17.1% were nurses.

**TABLE 4:** Characteristics of conjoint analysis respondents (*n* = 220).

		*n* or mean	(% or SD)
Age (years old)		52.8	(8.9)

Gender	Female	165	(75.0%)

Job title	Nurse	168	(76.4%)
	Physician	5	(2.3%)
	Administrative	28	(12.7%)
	Care worker	2	(0.9%)
	Other	17	(7.7%)

Position	Facility director equivalent	30	(13.6%)
	Facility deputy director/administrative director	19	(8.6%)
	Nursing director equivalent	97	(44.1%)
	Deputy nursing director equivalent	38	(17.3%)
	Nurse manager equivalent	18	(8.2%)
	Head nurse equivalent	5	(2.3%)
	Staff	10	(4.5%)
	Other	3	(1.4%)

Duration of employment at current facility (years)		17.7	(12.9)

Duration of employment duties (years)		5.3	(4.8)

Facility type	Hospital (over 400 beds)	45	(20.5%)
	Hospital (200–399 beds)	57	(25.9%)
	Hospital (less than 200 beds)	46	(20.9%)
	Clinic	10	(4.5%)
	Homecare nursing agency	27	(12.3%)
	Long‐term care facility	35	(15.9%)

Facility location	Metropolis or major city	50	(22.7%)
	City (prefectural capital)	33	(15.0%)
	City (nonprefectural capital)	118	(53.6%)
	Towns and villages	19	(8.6%)

Abbreviation: SD, standard deviation.

### 3.3. Conjoint Analysis: The Relative Importance of the Components of Nurses’ Employability

From the ranking data of the 16 virtual profiles (excluding the two holdout cases used for validation), the partial utility values and average relative importance of the factors were estimated according to the facility type, excluding the two holdout cases (Table 5). All facility types exhibited rank correlation coefficients above 0.90 (*p* < 0.01), indicating a strong correlation between estimations and respondents’ answers. Kendall’s rank correlation coefficient for holdout cases was 1.00, except for clinics, which was −1.00, indicating that the actual clinic ratings were the opposite of the estimated efficacy values. Therefore, clinical data could not derive a valid estimation model.

**TABLE 5:** Part‐worth utilities and mean relative importance of employability components by facility type.

Attribute	Level	Hospital (over 400 beds) (*n* = 45)		Hospital (200–399 beds) (*n* = 57)		Hospital (less than 200 beds) (*n* = 46)		Clinic (*n* = 10)		Home care nursing agency (*n* = 27)		Long‐term care facility (*n* = 35)	
		Part‐worths	Relative Importance (%)	Part‐worths	Relative Importance (%)	Part‐worths	Relative Importance (%)	Part‐worths	Relative Importance (%)	Part‐worths	Relative Importance (%)	Part‐worths	Relative Importance (%)
Reasonability for change	1	1.31	21.93	1.25	21.98	1.23	20.41	0.27	18.92	0.98	20.24	0.99	14.93
	2	−2.62		−2.38		−2.24		−1.76		−1.78		−1.50	
	3	1.31		1.13		1.01		1.49		0.80		0.51	

Sustained employment	1	0.73	7.63	0.61	8.21	0.61	8.25	0.88	8.02	0.40	7.69	0.47	7.34
	2	−0.73		−0.61		−0.61		−0.88		−0.40		−0.47	

Readiness	1	0.50	6.54	0.38	5.27	0.56	7.79	0.98	11.49	0.55	6.79	0.82	10.70
	2	−0.50		−0.38		−0.56		−0.98		−0.55		−0.82	

Practical skill	1	0.83	15.11	0.71	13.40	0.71	13.21	1.33	13.46	0.77	13.61	0.47	15.70
	2	0.18		0.29		0.40		−0.02		0.09		0.53	
	3	−1.01		−1.00		−1.11		−1.32		−0.86		−1.00	

Problem‐solving orientation	1	0.40	5.76	0.49	6.73	0.57	7.05	0.60	6.95	0.44	7.38	0.35	6.38
	2	−0.40		−0.49		−0.57		−0.60		−0.44		−0.35	

Alignment	1	0.34	6.62	0.69	8.35	0.73	8.39	1.00	10.06	0.72	9.59	0.52	6.97
	2	−0.34		−0.69		−0.73		−1.00		−0.72		−0.52	

Relationships	1	1.41	14.65	1.39	13.18	1.23	13.24	1.38	13.47	0.96	10.13	1.33	15.67
	2	−1.41		−1.39		−1.23		−1.38		−0.96		−1.33	

Commitment	1	0.88	8.71	1.01	9.95	1.09	10.30	0.93	7.88	1.08	11.76	1.27	12.75
	2	−0.88		−1.01		−1.09		−0.93		−1.08		−1.27	

Motivation	1	1.35	13.05	1.41	12.93	1.17	11.35	0.70	9.75	1.26	12.80	0.80	9.56
	2	−1.35		−1.41		−1.17		−0.70		−1.26		−0.80	

Constant term		7.97		8.01		8.02		8.10		8.06		8.14	

		*Value*	*(* *p* *value)*	*Value*	*(* *p* *value)*	*Value*	*(* *p* *value)*	*Value*	*(* *p* *value)*	*Value*	*(* *p* *value)*	*Value*	*(* *p* *value)*

Pearson’s *R*		1.00	(< 0.01)	1.00	(< 0.01)	1.00	(< 0.01)	1.00	(< 0.01)	1.00	(< 0.01)	1.00	(< 0.01)
Kendall’s Tau		0.95	(< 0.01)	0.97	(< 0.01)	0.98	(< 0.01)	0.90	(< 0.01)	0.93	(< 0.01)	0.95	(< 0.01)
Kendall’s Tau for holdout set		1.00	.	1.00	.	1.00	.	−1.00	.	1.00	.	1.00	.

*Note:* (1) Relative importance represents the mean relative importance. (2) Reasonability for change: reasonability for changing facility; sustained employment: experience of sustained employment in one organisation; readiness: readiness to work; practical skill: practical nursing skill; alignment: alignment towards nursing; relationships: establishing relationships; commitment: commitment to organisation; motivation: motivation for nursing. Level definitions: (1) reasonability for change: 1 = with, 2 = without and 3 = unknown. (2) Practical skill: 1 = excellent, 2 = moderate and 3 = poor. (3) All other attributes: 1 = with/good and 2 = without/poor.

Hospitals with more than 400 beds, 200–399 beds or fewer than 200 beds shared common patterns of relative importance. The top factor across all facility types was “Reasonability for changing facility” (20.41%–21.98%), followed by “Practical nursing skills”, “Establishing relationships with others” and “Motivation for nursing”. In homecare agencies, “Reasonability for changing facility” remained the most important (20.24%), with “Practical nursing skills” (13.61%) and “Motivation for nursing” (12.80%) following. Long‐term care facilities prioritised “Practical nursing skills” (15.70%) and “Establishing relationships with others” (15.67%), with “Reasonability for changing facility” in third (14.93%) and “Readiness to work” fifth (10.70%).

## 4. Discussion

The qualitative analysis of interviews with nurse recruiters and job placement agencies identified that nurses’ employability primarily centres on *the ability to work together on the nursing care that the organisation is trying to provide*. Key components of employability included the expectation of long‐term retention within the organisation; practical nursing skills; a positive approach to challenges; a willingness to learn nursing as both a profession and ideology required by the organisation; the ability to build strong relationships with patients, families and staff; a commitment to contribute meaningfully to the organisation’s improvement and intrinsic motivation for nursing. Furthermore, conjoint analysis revealed shared features and differences in the relative importance of these employability components across facility types.

The first point of the discussion addresses the characteristics of employability components that influence nurses’ interorganisational mobility. The employability characteristics of newly graduated nurses were conceptually distinct from those of experienced nurses, who are the focus of this study. Previous research on the essential skillsets of new graduate nurses has highlighted attributes such as “broad general knowledge”, “flexibility”, “problem‐solving abilities”, “work readiness”, “focus”, “integrity”, “teamwork” and “continuous critical thinking” [9], as well as “ability to execute care as planned”, “autonomous decision‐making”, “effective collaboration”, “commitment to professional development” and “competence in using medical equipment” [8]. These elements reflect the foundational knowledge and attitudes necessary for beginning a nursing career.

In contrast, the findings indicated that experienced nurses were perceived as demonstrating employability by seamlessly integrating into the organisation. The recruiters described them as possessing specific practical skills and interpersonal competencies that enabled smooth job performance. Given the substantial financial, time and human resource costs associated with training newly hired nurses [4, 26], employers are likely to prioritise hiring experienced nurses who can contribute immediately with minimal training.

Our findings also revealed that employers evaluate nurses’ abilities to fulfil their roles as organisational members based on their experience and specific competencies. Employability in this context includes an understanding of the nursing perspective within the organisation and commitment to its goals. These attributes are critical as nurses transitioning to a new workplace undergo an organisational resocialisation process [27]. Organisations impart information about their norms, values, cultures, work procedures and ethics to new employees during this process. This resocialisation may require nurses to reframe the tasks and attitudes acquired in previous workplaces, adapt to new expectations and embrace unfamiliar perspectives that may be unfamiliar. The emphasis on aligning new employees with organisational norms may partially reflect Japan’s cultural context, where group harmony and organisational commitment are highly valued [28].

In all hospitals and homecare nursing agencies, except for long‐term care facilities, the most critical factor was the clarity of applicants’ reasons for and objectives behind previous job changes. High nurse turnover imposes substantial financial and operational costs on healthcare facilities [1, 29]. Moreover, the turnover rate of experienced nurses exceeds that of newly graduated nurses [30]. Across all facility types, the potential for applicants to remain within the organisation for a meaningful duration was a key consideration. Past job changes and their underlying motivations indicated how likely applicants were to be willing to stay with the organisation long enough to support its stability and operational goals.

In hospitals, regardless of bed size, equal emphasis was placed on “Practical skill”, “Establishing relationships with others” and “Motivation for nursing”. This suggests that, irrespective of the facility’s capacity, experienced nurses are expected to possess the skills and readiness to contribute immediately contribute to employment.

Homecare nursing agencies emphasise that nurses possess unique characteristics, including practical skills, motivation for nursing, autonomy in decision‐making, variety in patient situations and the nurse’s role as a central figure in care, with the patient at the core of all activities [31]. These features highlight the specific demands and focus of homecare nursing. However, the high number of nursing respondents may have skewed the focus towards nursing identity in this study.

Although an analysis of the differences among the participant groups, including nurses, was not conducted because of the limited sample size of non‐nurse respondents, they were directly involved in hiring decisions for nurses. Thus, their perspectives likely reflect real‐world hiring practices and criteria. This lends credibility to the findings as a representation of the genuine recruitment decision‐making processes.

Practical skills and relationships with others are critical in long‐term care facilities. Unlike hospitals, these facilities require more frequent collaboration with other professionals, such as care workers. They also have responsibilities, such as developing nursing plans, sharing information with multidisciplinary teams and facilities and communicating with clients’ family members or key contacts [32]. Consequently, the ability to make autonomous decisions, maintain high performance levels and collaborate effectively collaborate with professionals, both within and outside the facility, is essential.

### 4.1. Limitations

This study has three primary limitations. First, because the research was conducted in Japan, the findings may strongly reflect cultural values unique to Japan, such as group harmony and collective decision‐making. These cultural factors could influence the recruitment priorities of participants and their perceptions of employability, possibly limiting the generalizability of the results to other cultural contexts.

Second, there is the potential for virtual bias, as virtual profiles may not entirely capture the actual decision‐making processes of respondents during nurse recruitment. Furthermore, the respondents’ characteristics might have influenced the results. The participants were increasingly interested in nurse recruitment, and the findings for each facility type reflected their unique professional perspectives.

Third, hiring decisions are not solely based on applicant profiles. Factors, such as interview performance and interpersonal impression, which were not considered in this study, may also influence recruitment decisions.

Despite these limitations, this study offers new insights into how nurse recruiters perceive employability and the factors that influence their selection preferences.

Furthermore, although the findings are grounded in Japan’s healthcare and cultural context, the underlying mechanisms identified—particularly the alignment between nurses’ professional orientation and organisational values in employability assessment—may also be relevant for healthcare systems that share similar organisational characteristics, such as emphasis on teamwork, organisational learning and sustained professional engagement.

### 4.2. Implications for Practice

The findings of this study have several practical implications.

First, the findings provide important insights for nurses seeking to enhance their employability according to workplace preferences. By understanding the unique expectations of different facility types, nurses can focus on developing skills and competencies that align with specific organisational needs. Additionally, nurse managers can play a crucial role in fostering employability among their staff and empowering nurses to build their careers independently. By understanding the factors that influence employability, they can more effectively support nurses in developing the necessary skills and attitudes to succeed in their organisations. This approach may ultimately enhance an organisation’s attractiveness as a workplace.

Furthermore, these results have implications for employers. By clarifying the employability components valued for each facility type, healthcare organisations can develop more accurate and informative job descriptions and recruitment materials. Tailoring job postings to reflect these priorities may improve the alignment between applicants’ strengths and organisational expectations, thereby reducing mismatches in hiring and supporting long‐term retention.

## 5. Conclusions

This study identified the key employability components for nurses transitioning between organisations and highlighted how these components vary depending on the type of facility used. Practical skills, motivation and adaptability to organisational norms are universally valued. However, specific demands may vary depending on the context.

In hospitals, emphasis is placed on practical skills, the ability to build relationships with others and motivation for nursing, reflecting the expectations of experienced nurses who can immediately contribute to clinical practice. Homecare nurses prioritised motivation for nursing, whereas long‐term care facilities emphasised building strong relationships with others.

These findings provide important insights for nurses seeking to enhance their employability according to workplace preferences. Future research should investigate practical methods for nurses to cultivate employability components and evaluate the outcomes of such developments in individual careers and organisational performance.

## Author Contributions

Tamaki Isobe conceived the study, designed the study, conducted data collection and analysis and drafted the manuscript. Yukie Takemura contributed to the study design, data interpretation and critical revision of the manuscript. Keiko Kunie contributed to data interpretation and critical revision of the manuscript.

## Funding

This study was funded by the JSPS KAKENHI (grant number: 20K10680), a joint research grant from FIRSTSTAR Healthcare Inc., and a research grant from the Japan Academy of Nursing Administration and Policies.

## Disclosure

All authors approved the final version of the manuscript. Funding sources were not involved in the study design, data collection, analysis, interpretation or writing of the manuscript. A portion of this study was presented at the 25th Annual Conference of the Japan Academy of Nursing Administration and Policies (August 2021, online) and the 26th Annual Conference of the Japan Academy of Nursing Administration and Policies (August 2022, Fukuoka).

## Conflicts of Interest

The authors declare no conflicts of interest.

## Data Availability

Data supporting the findings of this study are available upon request from the corresponding author. The data are not publicly available because of privacy and ethical restrictions.
